# Changes of Brain Functional Connectivity in End-Stage Renal Disease Patients Receiving Peritoneal Dialysis Without Cognitive Decline

**DOI:** 10.3389/fmed.2021.734410

**Published:** 2021-11-24

**Authors:** Ting-Yu Chang, Hsin-Hsu Wu, Yi-Jung Li, Ho-Ling Liu, Chih-Hua Yeh, Hui-Shan Jian, Kuo-Lun Huang, Tsong-Hai Lee, Ya-Chung Tian, Changwei W. Wu

**Affiliations:** ^1^Department of Neurology, Stroke Section, Chang Gung Memorial Hospital, Linkou Medical Center and College of Medicine, Chang Gung University, Taoyuan, Taiwan; ^2^Graduate Institute of Clinical Medical Sciences, Chang Gung University, Taoyuan, Taiwan; ^3^Kidney Research Center, Department of Nephrology, Chang Gung Memorial Hospital, Linkou Medical Center and College of Medicine, Chang Gung University, Taoyuan, Taiwan; ^4^Department of Imaging Physics, The University of Texas M.D. Anderson Cancer Center, Houston, TX, United States; ^5^Department of Medical Imaging and Intervention, Chang Gung Memorial Hospital, Taoyuan City, Taiwan; ^6^Graduate Institute of Mind, Brain and Consciousness, Taipei Medical University, Taipei, Taiwan; ^7^Brain and Consciousness Research Center, Shuang-Ho Hospital-Taipei Medical University, New Taipei, Taiwan

**Keywords:** end-stage renal disease, peritoneal dialysis, resting-state functional MRI, functional connectivity, graph theory, default-mode network, cognitive function

## Abstract

**Background:** Functional connectivity detected by resting-state functional MRI (R-fMRI) helps to discover the subtle changes in brain activities. Patients with end-stage renal disease (ESRD) on hemodialysis (HD) have impaired brain networks. However, the functional changes of brain networks in patients with ESRD undergoing peritoneal dialysis (PD) have not been fully delineated, especially among those with preserved cognitive function. Therefore, it is worth knowing about the brain functional connectivity in patients with PD by using R-fMRI.

**Methods:** This case-control study prospectively enrolled 19 patients with ESRD receiving PD and 24 age- and sex- matched controls. All participants without a history of cognitive decline received mini-mental status examination (MMSE) and brain 3-T R-fMRI. Comprehensive R-fMRI analyses included graph analysis for connectivity and seed-based correlation networks. Independent *t-*tests were used for comparing the graph parameters and connectivity networks between patients with PD and controls.

**Results:** All subjects were cognitively intact (MMSE > 24). Whole-brain connectivity by graph analysis revealed significant differences between the two groups with decreased global efficiency (E_glob_, *p* < 0.05), increased betweenness centrality (BC) (*p* < 0.01), and increased characteristic path length (L, *p* < 0.01) in patients with PD. The functional connections of the default-mode network (DMN), sensorimotor network (SMN), salience network (SN), and hippocampal network (HN) were impaired in patients with PD. Meanwhile, in DMN and SN, elevated connectivity was observed in certain brain regions of patients with PD.

**Conclusion:** Patients with ESRD receiving PD had specific disruptions in functional connectivity. In graph analysis, E_glob_, BC, and L showed significant connectivity changes compared to the controls. DMN and SN had the most prominent alterations among the observed networks, with both decreased and increased connectivity regions. Our study confirmed that significant changes in cerebral connections existed in cognitively intact patients with PD.

## Introduction

End-stage renal disease (ESRD) causes a severe burden on global public health and has a poor prognosis. According to a recent report (1), the Taiwanese population has the highest prevalence of ESRD with dialysis therapy in the world. Asides from systemic complications, neurological manifestations are common in the ESRD population. Studies over the past decade showed that when compared with healthy individuals, patients with ESRD had a higher incidence of stroke (2), cognitive impairment, and dementia, regardless of their renal replacement modalities (3, 4).

The underlying mechanism of higher incidence of cerebral diseases in patients with ESRD, the so-called “kidney–brain axis,” is complicated. Based on the similarity of anatomical and vasoregulatory features of the brain and kidneys, vasculopathy of cerebral and glomerular small vessels could be the common pathogenesis (5, 6). Previous magnetic resonance imaging (MRI) studies discovered brain structural changes in patients with ESRD. Compared with the age-matched normal subjects, patients with ESRD had more white matter lesions (7), silent infarcts, cerebral atrophy (8), and axonal demyelination (9). Beyond the structural alterations, resting-state functional MRI (R-fMRI) recently has become a valuable tool in discovering functional changes of brain networks in patients with ESRD.

Resting-state functional MRI is based on the blood oxygen level-dependent (BOLD) principle at an intrinsic spontaneous (resting) status, where the R-fMRI signals can be analyzed to detect the temporal synchronizations across distant brain areas, referred to as functional connectivity (10). The alterations of functional connectivity in patients with ESRD may present with an extensive and variable pattern based on different analytical algorithms. Earlier, R-fMRI studies of patients with ESRD found decreased brain regional activity in a diffuse pattern over bilateral frontal, parietal, and temporal lobes, or impaired default-mode network (DMN) connectivity in the posterior cingulate cortex, precuneus, and medial prefrontal cortex (11, 12), without mention of dialysis modality. Subsequent studies have identified different aspects of connectivity reduction in patients with ESRD (13–16). Zheng et al. observed the decreased connectivity to be predominantly related to the prefrontal lobe in patients with ESRD receiving hemodialysis (HD), which involves planning complex cognitive behavior (13). Chen et al. found that patients with ESRD with HD had decreased regional homogeneity (ReHo) in DMN when compared to those without replacement therapy (14). Evidence on examining the global functional connectivity of ESRD patients with HD found abnormal intrinsic dysconnectivity pattern of salience network (SN) regions over bilateral insula and dorsal anterior cingulate cortex (15). However, most of the above ESRD studies were restricted to patients with HD with a certain extent of cognitive impairment.

The procedure of HD itself may cause significant systemic circulatory stress and cardiac stunning (17, 18). This repetitive stress may lead to persistent perfusion anomalies and accelerate end organ damage, including the brain (19, 20). Unlike HD, the peritoneal dialysis (PD) procedure does not involve extracorporeal circulation, and patients with PD have a less hemodynamic impact based on their daily dialysis. Very few brain fMRI studies focused on patients with PD only and showed inconsistent results at the current stage. One previous study discovered that patients undergoing PD had lower values of the amplitude of low-frequency fluctuations (ALFF) in the parietal lobes and left precuneus (21). Recently, another study demonstrated that the patterns of functional connectivity among patients with PD and HD were not consistent. Compared to the healthy controls, only patients with HD but not patients with PD had significant changes in global functional connectivity. The authors concluded that the brain networks may be affected by different types of renal replacement therapy (22). Moreover, disruption of brain function may precede neurological symptoms. Hence, we aimed at assessing changes of connectivity in cognitively-intact patients with ESRD under regular PD therapy. We believed that recognizing connectivity change of neurologically asymptomatic patients with PD might help to detect subtle brain dysfunctions and identify susceptible brain regions in this specific group. Technically, we took two analytical strategies, global and local searches, to comprehensively present the altered integrity of brain functionality among patients with PD. Regarding the global search, the graph analysis was adopted to reveal the functional alterations of the whole-brain connectivity (23). In the local search, network-specific connectivity maps were analyzed to reveal the functional changes of multiple ESRD-related networks, such as DMN and salience network (SN) (12, 15). Furthermore, we also included the sensorimotor network (SMN) for the reported slow motor performances and the hippocampal network (HN) for the memory disturbances in the patients with ESRD on PD therapy (4, 21).

## Materials and Methods

### Ethics Statement

Written informed consent was obtained from all subjects. This study was conducted in accordance with the principles expressed in the Declaration of Helsinki and was approved by the Institutional Review Board of Chang Gung Memorial Hospital (IRB approval number: 102-1346A3).

### Patients and Clinical Evaluation

This cross-sectional study recruited 19 patients with ESRD (including nine men) who received PD for at least 1 year and 24 age- and sex-matched controls (including nine men) with normal renal functions. All participants were right-handed. The causes of ESRD in the 19 patients included diabetic nephropathy in six patients, hypertensive nephropathy in five, chronic glomerulonephritis in four, renal artery stenosis in one, kidney graft failure in one, and unknown etiology in two patients. We excluded patients with known neurological disorders and psychiatric diseases, and those taking any medication involving the central nervous system (e.g., sleeping pills). Each subject underwent pain assessment with the numeric scales and depression screening based on Patient Health Questionnaire-2 (PHQ-2) by a neurologist (T.Y.C.) at the outpatient clinic before the enrollment. Baseline mini-mental status examination (MMSE) score was evaluated by a licensed psychologist. Subjects with any evidence of pain, depressive mood, or with an MMSE score below 24 were also excluded (24, 25). A routine laboratory test for the patients was done within 1 week prior to performing the MRI scan. For the controls, basic biochemistry data was recorded upon enrollment. Each participant received compensation of ~US $20.

### Image Acquisition

Magnetic resonance imaging was conducted on a 3-T scanner (GE Healthcare, Milwaukee, WI) with T1-weighted images, T2-weighted fluid-attenuated inversion recovery (FLAIR) image, and R-fMRI. The T1-weighted image was performed using the BRAVO sequence with 512 × 512 × 160 matrix size; 0.5 × 0.5 × 1 mm resolution; inversion time = 450 ms; repetition time (TR) = 8,252 ms; echo time (TE) = 3.2 ms; flip angle (FA) = 12°; and NEX = 1. The T2-FLAIR sequence was performed to exclude anatomical lesions, especially the white matter hyperintensities, by using the following parameters: slice thickness = 3.5 mm; 512 × 512 × 32 matrix size; field of view (FOV) = 220 × 220 mm; TR = 9,000 ms; TE = 140 ms; inversion time (TI) = 2,250 ms; and FA = 90°. R-fMRI was performed using a ${\text{T}}_{2}^{*}$-weighted single-shot gradient-echo echo-planar imaging sequence (TR/TE/FA = 2,000 ms/30 ms/90°, FOV = 220 mm × 220 mm; in-plane matrix = 64 × 64; in-plane resolution = 3.4 × 3.4 mm, and slice thickness = 4 mm). For each patient, 36 continuous axial slices per volume and 180 volumes were acquired with an acquisition time of 6 min. During R-fMRI scanning, the subjects were asked to keep their eyes closed, maintain head position, not fall asleep, and think about nothing in particular.

### Data Preprocessing

Resting-state functional MRI data preprocessing was conducted using SPM8 (Welcome Department of Cognitive Neurology, Institute of Neurology, London, UK) and REST (26) in MATLAB (Mathworks, Natick, MA, USA) platform. All functional datasets underwent slice timing correction, realignment for head motion correction, spatial normalization to the Montreal Neurological Institute space (re-sampled to 2 × 2 × 2 mm^3^ of voxel size), and spatial smoothness with full-width half-maximum of 8 mm. Head motion exceeding ±3 mm of translation or ±0.5 mm framewise displacement were excluded from the dataset. Subsequently, the linear trend was removed. Physiological noise from the CSF and white matter was regressed out, and also the motion parameters of maximum translation and framewise displacement were regressed out in each dataset [the maximum translation (mean ± SD): Controls = 0.81 ± 0.63 mm, PD = 1.05 ± 0.58 mm, *non-significance*; and the averaged framewise displacement (mean ± SD): Controls = 0.12 ± 0.07 mm, PD = 0.16 ± 0.08 mm, *non-significance*]. Finally, the data were band-pass filtered at the frequency band of 0.01–0.08 Hz.

### Global Connectivity: Graph Analysis

Graph theory analysis constructs the brain regions (“nodes”) and the connections between the regions (“edges”) as a complex network, referred to as “connectivity,” in a topological organization (27). To estimate the global change of brain networks, the pre-processed R-fMRI dataset was parcellated by the well-defined automated anatomical labeling (AAL) atlas into 90 regions (nodes) of interest for each patient (28) (see Supplementary Table 1). The mean time series of each region was then obtained by averaging the signal intensities over all voxels within a region of interest. After calculating the Pearson correlation coefficient (*r*) between each pair of 90 regions of interest, a 90 × 90 correlation matrix was constructed for each individual. Cross-region correlation coefficients were regarded as unidirectional edges if *r* values exceeded a threshold. Subsequently, specific *r* thresholds were chosen to match fixed density values (0.1, 0.2, and 0.3), where the density was defined as connected edges divided by the maximum number of possibly connected edges over the entire brain. The density range of 0.1–0.3 was found to be reliable in the R-fMRI literature (29). Thus, a binary connectivity matrix was generated after the *r* thresholding, and the graph analysis was applied to the binary matrix to estimate the whole-brain network properties using brain connectivity toolbox (30). The node-based network properties included degree, clustering coefficient (C), local efficiency (E_loc_), and betweenness centrality (BC), and the global network properties included characteristic path length (L), global efficiency (E_glob_), modularity (Q), small-worldness (σ), and assortativity coefficient (A). Supplementary Figure 1 shows the topological parameters of graph theory used in this study. Mathematical definitions of these parameters are according to the study by Rubinov and Sporns (30), and the brief descriptions and illustrations of the meanings of these parameters are provided in Supplementary Material.

### Network-Specific Connectivity: Seed-Based Analysis

As the large AAL regions could dilute the temporal coherence and minimize the spatial specificity, we further adopted the seed-based analysis to measure the functional alterations of the four brain networks. Spherical seeds with a radius of 4 mm were prescribed from four target networks for functional evaluations: the DMN [seed at posterior cingulate cortex: (0, −51, 30)], the SMN [seed at right precentral gyrus: (36, −25, 57)], the SN [seed at right insula: (30, 9, 10)], and the HN [seed at right anterior hippocampi: (24, −20, −22)] (31). Time courses averaged within the seeds were used as a reference to perform correlation analysis over the entire brain. The resulting correlation coefficients were converted using Fisher's *z*-transform, resulting in the connectivity map for the subsequent group analysis.

### Intrinsic Brain Activity: Amplitude of Low-Frequency Fluctuations

Beyond the connectivity changes, we also analyzed additional R-fMRI index of intrinsic brain activity, ALFF, in both patient and control groups. The related details are provided in Supplementary Material.

### Statistical Analysis

Demographic and clinical data between the two groups were compared using the chi-squared test or independent *t*-test. An independent *t*-test was used for comparing the graph analysis parameters and connectivity networks between the two groups. Linear regression and Pearson correlation coefficient *r* were applied for evaluating the relationship between clinical data and graph parameters. These statistical analyses were performed using the Statistical Package for Social Sciences [IBM SPSS Statistics for Mac, version 24], and the results were considered significant at *p* < 0.05. For the group comparison of connectivity maps, we used AFNI software (32). Considering multiple corrections in fMRI results, the one-sample group average was displayed with family-wise error (FWE)-corrected *p* < 0.05 with cluster threshold of 20 voxels, whereas the between-group contrast maps were demonstrated with 3dClustSim-corrected *p* < 0.05. The multiple comparisons in the group analysis were corrected through the 3dClustSim approach with the auto-correlation function, and the significance level was *p* < 0.05 (uncorrected *p* < 0.001, cluster threshold = 103 voxels).

## Results

### Demographic Data

Table 1 shows the demographic characteristics of the two groups. The education years of patients and the age- and sex-matched controls were 13.4 ± 3.3 years vs. 11.1 ± 4.3 years (*p* = 0.06), respectively, and the two groups had similar MMSE performance (28 vs. 28.9, *p* = 0.13), which represented no clinically overt cognitive impairment. Among the patient group, the average duration of receiving PD was 4.2 ± 3.7 years. The PD duration for each patient is listed in Supplementary Table 2. As hypertension and diabetes mellitus (DM) could influence intrinsic brain activities (33, 34), to account for this issue, we enrolled controls and patients with ESRD with similar proportions of chronic diseases. The subjects from both groups had comparable baseline characteristics among the percentage of major systemic diseases (hypertension, coronary artery disease, and DM), glycated hemoglobin (HbA1C), and lipid profiles. Compared to the controls, the patients had a higher level of uric acid (7.0 ± 0.7 vs. 6.1 ± 1.6, *p* = 0.02). Data of white blood cell count, hemoglobin, hematocrit, and albumin were only available for the patient group.

**Table 1:** Demographic characteristics of subjects.

**Characteristics**	**PD (*n* = 19)**	**Controls (*n* = 24)**	***P***
Sex, male (%)	47.4	37.5	0.5^a^
Age (mean ± SD)	49.8 ± 13	51.2 ± 7.6	0.6^a^
Education, years (mean ± SD)	13.4 ± 3.3	11.1 ± 4.3	0.06^a^
Duration of PD, years (mean ± SD)*	4.2 ± 3.7	N/A	N/A
MMSE score	28.2 ± 1.5	28.9 ± 1.6	0.1^a^
Cause of ESRD	Variable**	N/A	N/A
Chronic illness and personal history, presence [*n*, (%)]			
Hypertension	16/19 (84)	14/24 (58)	0.07^b^
Hyperlipidemia	4/19 (21)	4/24 (17)	0.99^b^
CAD	2/19 (11)	2/24 (8)	0.81^b^
DM and pre-DM^+^	8/19 (42)	8/24 (33)	0.56^b^
Alcohol^++^	0/19 (0)	0/24 (0)	1.0^b^
Laboratory data (mean ± SD)			
White blood cell count, 10^12^/L	7.5 ± 1.7	N/A	N/A
Hemoglobin, g/dl	10.7 ± 0.9	N/A	N/A
Hematocrit, %	31.7 ± 2.7	N/A	N/A
Albumin, g/dl	3.8 ± 0.4	N/A	N/A
Uric acid, mg/dl	7.0 ± 0.7	6.1 ± 1.6	0.02^a^, ^#^
HbA1C, %	5.9 ± 0.8	5.5 ± 0.5	0.13^a^
Total cholesterol, mg/dl	175 ± 27.8	186 ± 36.3	0.29
LDL-cholesterol, (mg/dl)	119 ± 26.7	105 ± 32.2	0.22
HDL-cholesterol (mg/dl)	45 ± 13.9	51 ± 14.7	0.16
Triglyceride (mg/dl)	141 ± 59.9	144 ± 99.9	0.89

*The demographic comparisons between the two groups:*

a *independent t-test;*

b *chi-square test*.

*Significance is defined when p < 0.05*.

# *p < 0.05*.

* *The duration of undergoing dialysis for each patient is listed in Supplementary Material*.

** *Cause of ESRD: total 19 patients with ESRD, including 6 with diabetic nephropathy, 5 with hypertensive nephropathy, 4 with chronic glomerulonephritis, 1 with renal artery stenosis, 1 with graft failure, and 2 with unknown etiology*.

+ *pre-DM: defined as HbA1C > 5.7%*.

++ *Alcohol: alcohol use more than “drinking in moderation” (defined as <2 drinks in a day by the “Dietary Guidelines for Americans 2020–2025,” U.S. Department of Health and Human Services and U.S. Department of Agriculture)*.

*PD, peritoneal dialysis; MMSE, mini-mental status examination; ESRD, end-stage renal disease; CAD, coronary artery disease; DM, diabetes mellitus; N/A, not applicable*.

### Graph Analysis

**Figure 1:**
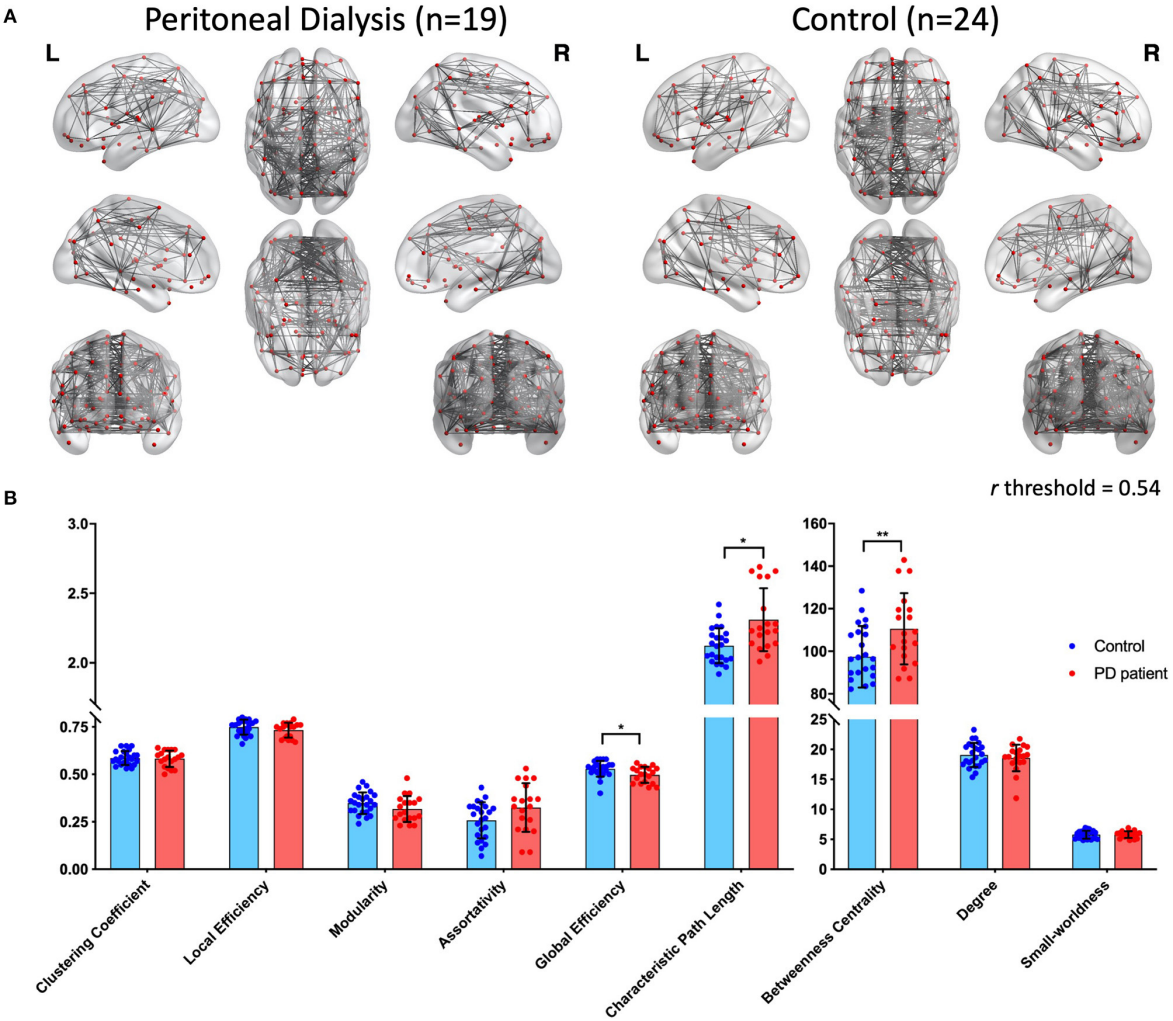
**(A)** Whole-brain connections by graph analysis of patients receiving peritoneal dialysis (left) and controls (right) (density = 0.2). Nodes were chosen from the 90 regions in the automated anatomical labeling (AAL) template, and the edges are shown under the condition of a threshold above 0.54. **(B)** Distributions of nine parameters from graph analysis. Blue dots represent controls, and red dots represent patients with PD. The columns and error bars represent the means and SD, respectively. There are significant differences between the two groups in global efficiency, characteristic path length, and betweenness centrality. PD, peritoneal dialysis; *p < 0.05; **p < 0.01.

The AAL-based edges are shown in Figure 1A (density = 0.2) for both groups. Overall, the connections (edges) were denser in the control group when compared to the PD group. Table 2 and Figure 1B demonstrate the nine network properties from graph analysis. Compared with the controls, the patients with ESRD had stronger BC (PD: 110.54 ± 16.71 vs. controls: 97.39 ± 14.45, *p* < 0.01) and L (PD: 2.31 ± 0.23 vs. controls: 2.12 ± 0.13, *p* < 0.01). Meanwhile, the controls had higher E_glob_ than the patients (PD: 0.50 ± 0.04 vs. controls: 0.53 ± 0.04, *p* < 0.05).

**Table 2:** Comparison of parameters from graph analysis.

**Density = 0.2 Parameters**	**PD (*n* = 19) (mean ± SD)**	**Control (*n* = 24) (mean ± SD)**	***p*-value^+^**
Degree	18.58 ± 2.22	19.09 ± 2.01	0.443
Clustering coefficient	0.58 ± 0.04	0.59 ± 0.04	0.766
Local efficiency	0.73 ± 0.04	0.75 ± 0.04	0.191
Betweenness centrality	110.54 ± 16.71	97.39 ± 14.45	0.008**
Characteristic path length	2.31 ± 0.23	2.12 ± 0.13	0.001**
Global efficiency	0.50 ± 0.04	0.53 ± 0.04	0.019*
Modularity	0.32 ± 0.07	0.35 ± 0.06	0.116
Small-worldness	5.80 ± 0.55	5.79 ± 0.66	0.945
Assortativity	0.33 ± 0.13	0.26 ± 0.10	0.054

+ *Independent t-test was used for between-group comparisons. Significance is defined when p <0.05*.

* *p <0.05*.

** *p < 0.01*.

### Seed-Based Connectivity

**Figure 2:**
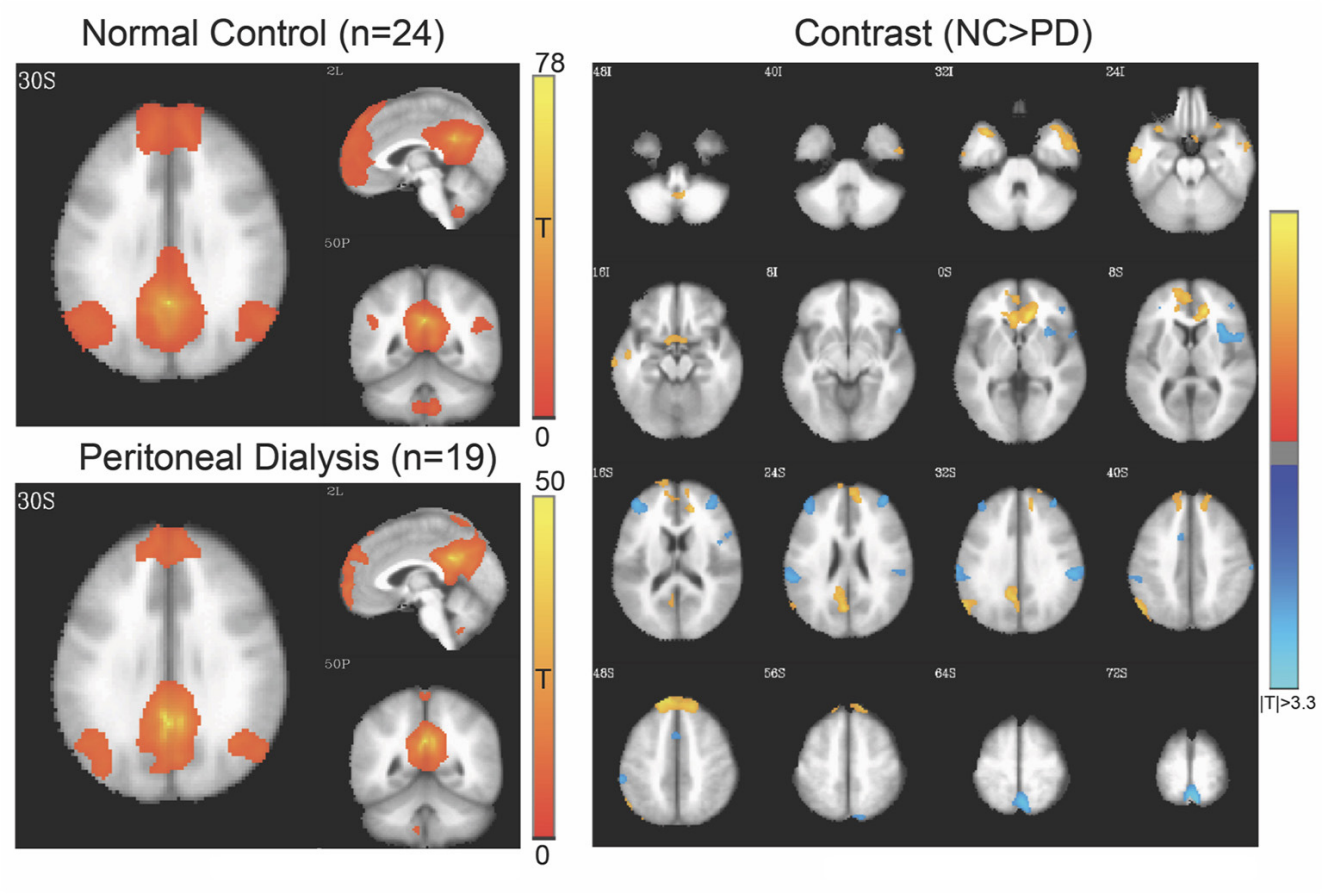
Default-mode network (DMN) of controls and patients with PD. Connectivity map of either group is shown at left side (FWE-corrected *p* < 0.05), and the contrast at right side (3dClustSim-corrected *p* < 0.05). In the contrast map, the orange color represents significantly increased connectivity in controls, including anterior cingulate gyrus, superior and medial frontal gyrus, temporal lobe, and precuneus, whereas the blue color represents decreased connectivity in controls, including the insula, inferior parietal lobule, middle frontal gyrus, and supplemental motor area. PD, peritoneal dialysis; NC, normal controls.

**Figure 3:**
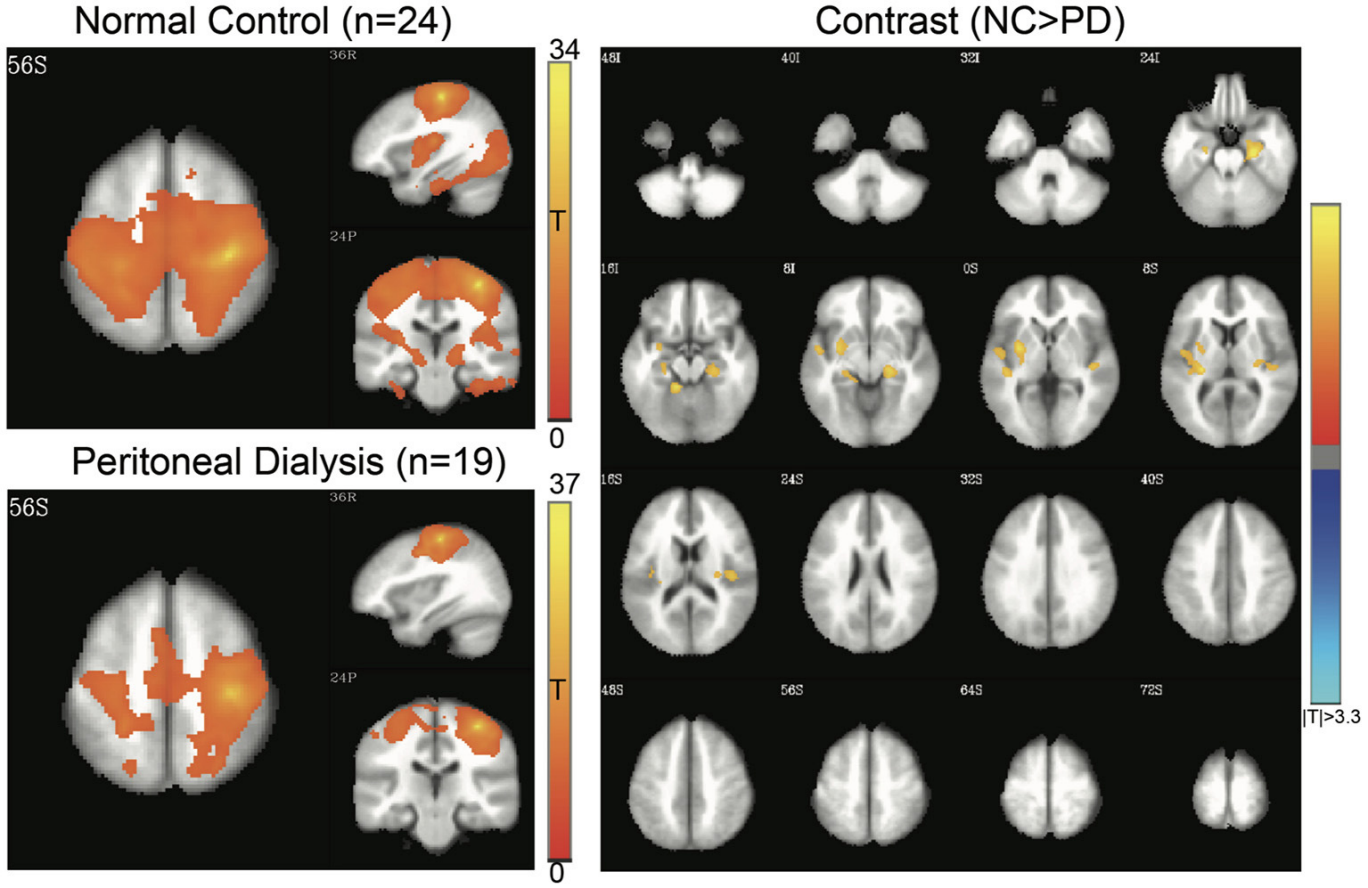
Sensorimotor network (SMN) of controls and patients with PD. Connectivity map of either group is shown at the left side (FWE-corrected *p* < 0.05), and the contrast map at the right side (3dClustSim-corrected *p* < 0.05). In the contrast map, the orange color represents significantly increased connectivity in controls, including parahippocampal gyrus and insula. PD, peritoneal dialysis; NC, normal controls.

**Figure 4:**
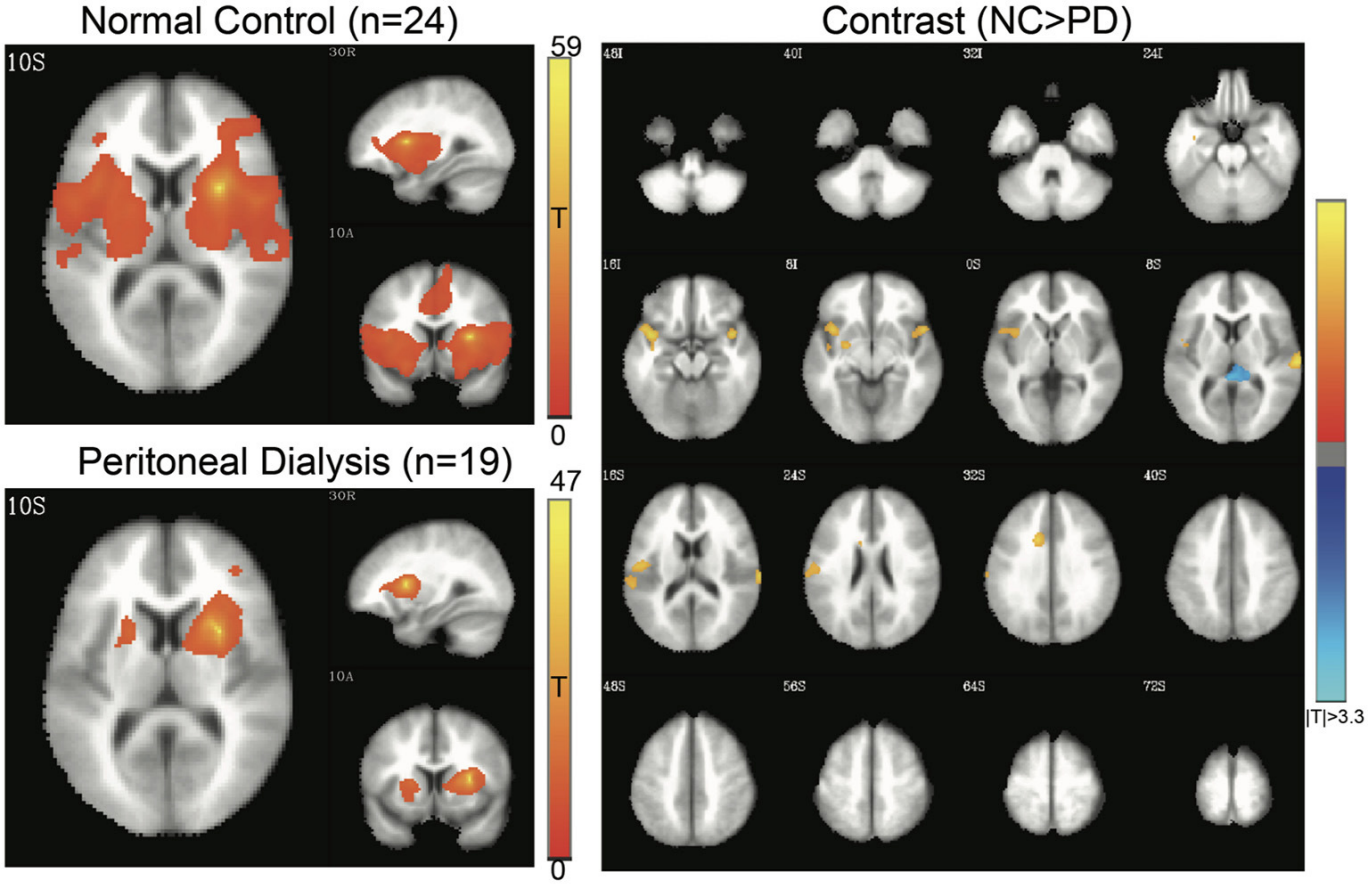
Salience network (SN) of controls and patients with PD. Connectivity map of either group is shown at the left side (FWE-corrected *p* < 0.05), and the contrast map at the right side (3dClustSim-corrected *p* < 0.05). In the contrast map, the orange color represents significantly increased connectivity in controls, including temporal gyrus, cingulate gyrus, and postcentral gyrus, whereas the blue color represents decreased connectivity in controls, mainly in the thalamus. PD, peritoneal dialysis; NC, normal controls.

**Figure 5:**
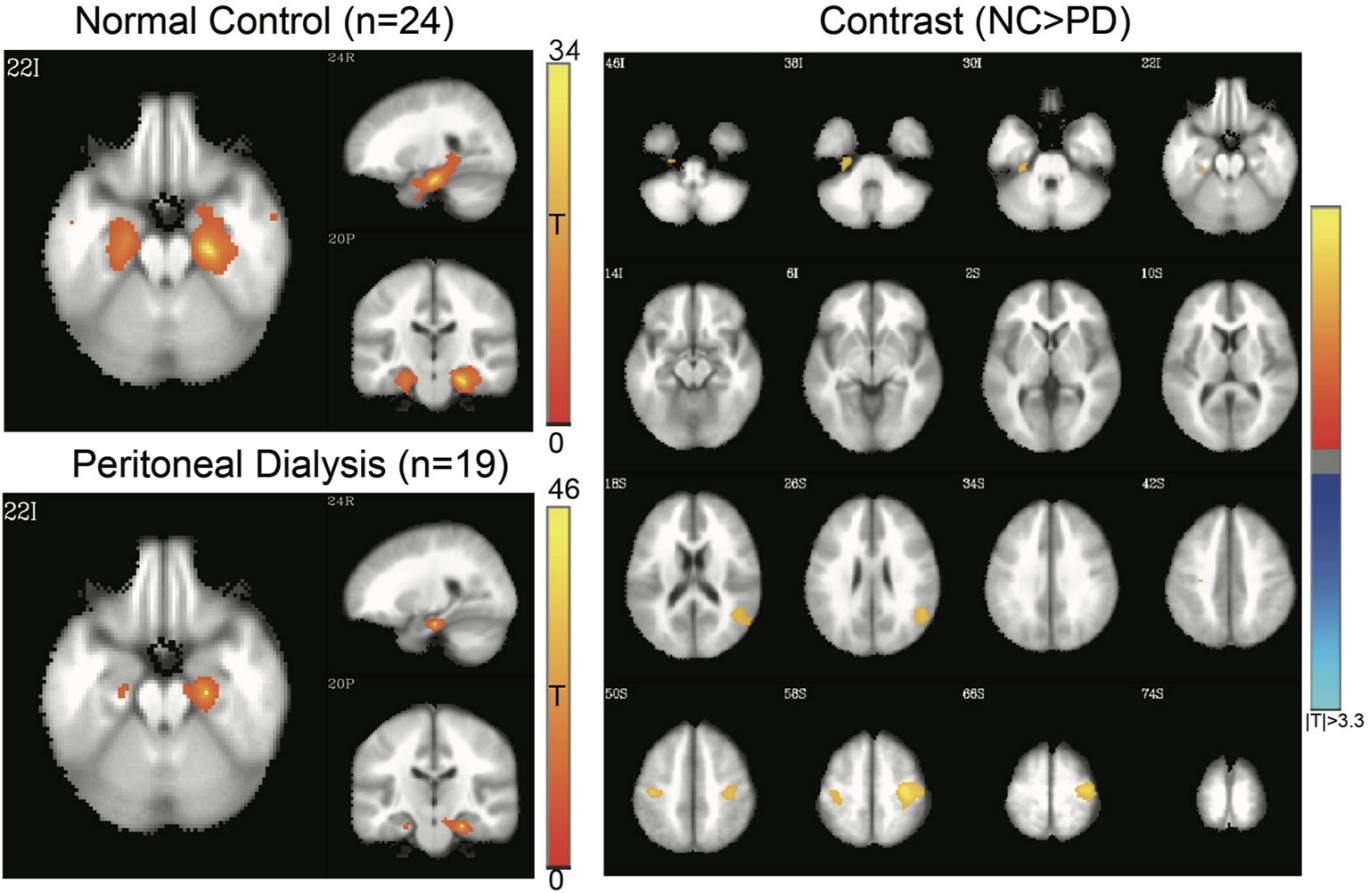
Hippocampal network (HN) of controls and patients with PD. Connectivity map of either group is shown at the left side (FWE-corrected *p* < 0.05), and contrast map at the right side (3dClustSim-corrected *p* < 0.05). In the contrast map, the orange color represents significantly increased connectivity in controls, whereas the blue color represents decreased connectivity in controls. PD, peritoneal dialysis; NC, normal controls.

Four networks were chosen for functional alterations in patients with ESRD through the seed-based connectivity analysis, including DMN (Figure 2), SMN (Figure 3), SN (Figure 4), and HN (Figure 5). The brain areas with significant differences indicated that their functional connection with the seed region changed in the patient group when compared with the controls. The regional details of the connectivity changes are described below and summarized in Table 3.

**Table 3:** Brain regions with significant connectivity differences between PD and controls of selected functional networks.

	**x (mm)**	**y (mm)**	**z (mm)**	**T**	**Cluster size (voxel)**	**Localization**
**DMN**						
Controls>PD^+^	10	32	2	5.93	738	Anterior cingulate
	−2	−68	28	5.85	591	Precuneus
	−14	50	48	5.48	1,824	Superior frontal gyrus
	−8	6	−18	4.66	221	Medial frontal gyrus
	−58	−66	30	5.28	372	Superior temporal gyrus
	52	2	−30	4.82	441	Middle temporal gyrus
	−34	14	−32	4.73	178	Superior temporal gyrus
	−58	−10	−26	4.69	342	Inferior temporal gyrus
PD>Controls^++^	36	14	6	4.5	575	Insula
	60	−30	32	4.4	338	Inferior parietal lobule
	−42	50	20	4.36	495	Middle frontal gyrus
	0	14	46	3.91	143	Supplemental motor area
**SMN**						
Controls>PD^+^	26	−18	−24	5.17	526	Parahippocampal gyrus
	−12	−40	−14	5.07	328	Parahippocampal gyrus
	−28	−24	10	4.83	1,042	Insula
	42	−20	14	4.22	346	Insula
**SN**						
Controls>PD^+^	42	8	−18	5.58	205	Superior temporal gyrus
	−42	8	−16	5.09	620	Superior temporal gyrus
	−14	14	34	4.7	165	Cingulate gyrus
	−50	−14	18	4.4	509	Postcentral gyrus
PD>Controls^++^	−2	−36	6	4.4	219	Thalamus
**HN**						
Controls>PD^+^	34	−20	60	5.36	998	Precentral gyrus
	−22	−20	−34	5.2	245	Parahippocampal gyrus
	−40	−22	50	4.36	286	Postcentral gyrus
	50	−54	24	4.33	424	Superior temporal gyrus

*PD, peritoneal dialysis; T, peak t-value; DMN, default-mode network; SMN, sensorimotor network; SN, salience network; HN, hippocampal network (p < 0.05, 3dClusterSim-corrected)*.

+ *Controls > PD: significantly bolder connectivity of normal controls*.

++ *PD > Controls: significantly bolder connectivity of patients with peritoneal dialysis*.

#### DMN

Figure 2 shows the DMN connectivity maps of the patients and controls. Compared with the controls, the patients with ESRD had lesser functional connectivity, mainly at the temporal gyri, anterior cingulate cortex, superior frontal gyrus, and precuneus; meanwhile, they also showed increased connectivity at the insula, middle frontal gyrus, and inferior parietal lobe.

#### SMN

In SMN (Figure 3), the patients had decreased functional connectivity at the insula and parahippocampal gyrus.

#### SN

In SN (Figure 4), the patients had significantly increased functional connectivity at the thalamus and decreased connectivity at the superior temporal gyrus, anterior cingulate gyrus, and postcentral gyrus when compared to controls.

#### HN

The HN of the patients and controls is shown in Figure 5. The controls had stronger functional connectivity than the patients in the precentral gyrus, postcentral gyrus, superior temporal gyrus, and parahippocampal gyrus.

### Correlation Between Uric Acid and the Global Connectivity

Since the uric acid levels showed significant differences between the patients and the controls, correlation analysis was performed to evaluate the relationship between uric acid and selected global connectivity metrics in the patient group and in all subjects. Linear regression by Pearson correlation coefficient showed no significant correlation between uric acid and global connectivity of BC, L, and E_glob_, either in the patient group or in all subjects. The correlation data is summarized in Supplementary Table 3.

### The Amplitude of Low-Frequency Fluctuations

The comparison of ALFF between both groups revealed no significant differences. The corresponding result is shown in Supplementary Figure 2.

## Discussion

We examined the brain connectivity of cognitively intact patients with ESRD undergoing PD and compared it with age- and sex-matched controls. Our findings suggested that patients with PD had functional connectivity profiles that were significantly different from controls, based on both graph analysis and seed-based network analysis. To the best of our knowledge, this is the first brain connectivity report including both graph theory analysis and seed-based networks data in patients with PD. Unlike HD, in which intradialytic hypotension is very common, patients with PD have less hemodynamic stress during their routine dialysis therapy (35). The disruption of functional connectivity in our patients might not be mainly caused by hemodynamic alterations.

In the review of literature, there were two R-fMRI studies focusing on patients with ESRD undergoing PD. The first study enrolled patients with impaired cognitive performance and the R-fMRI analysis used ALFF as the only indicator of network changes (21). Compared to the healthy controls, patients with PD showed lower ALFF values in the bilateral frontal and parietal lobes, especially in the left precuneus, left inferior parietal lobe, and left superior parietal lobe. The other study performed graph theoretical analysis on both structural and functional connectivity. In global connectivity, patients with PD only showed decreased “structural” connectivity but not “functional” connectivity, while in local connectivity, patients with PD had variable changes (including increased and decreased connectivity) in BC over right frontal operculum, right inferior temporal, right temporal cortex pole, right occipital pole, right supracalcarine, left insular cortex, and left postcentral region (22). It was noted that most patients with PD in that study also had cognitive impairment, especially in frontal/executive function. In our study, all subjects maintained good daily functions without clinical cognitive decline, and the comprehensive analysis of spontaneous brain activities helped to reveal subtle changes in the brain circuits in patients with PD. Instead of referring to specific regions, our graph analysis of the functional connections was examined in a general view, and the results showed increased BC, increased L, and decreased E_glob_ in the PD group. BC denotes the influence of a “node” (a brain region) on the flow of information between all other nodes (36). Stronger BC implies the enrichment of hub-like nodes in the brain of patients (i.e., multiple nodes control the information flow, so the connecting structure tends to be dispersed). The path length, L, is the average of all the shortest paths between all the node pairs in the brain, whereas the inverse measurement of L is E_glob_ (30). The high L value in the patients with PD insinuates relative estrangement between the nodes within the network, leading to potential inefficiency in regional communication. This was also evident in low E_glob_ in the patients, indicating the impaired ability of a network to communicate globally. Briefly, functional connectivity on graph analysis in our ESRD PD patients tended to show increased hub-nodes (higher BC), more distant connections between the nodes (longer L), and less efficient global communication (lower E_glob_). We suggested that the above graph metrics could be indicators for identifying global connectivity alterations in patients with PD.

The seed-based analysis for functional connectivity investigates the temporal synchronizations between brain regions (37), and the four networks of DMN (encompassing medial prefrontal cortex, posterior cingulate cortex, bilateral inferior parietal cortex, and bilateral superior temporal lobes) (31, 38), SMN (encompassing bilateral primary motor cortex and supplementary motor area) (39), SN (encompassing dorsal anterior cingulate cortex and bilateral anterior insula) (15), and HN (encompassing bilateral hippocampi) (40) are commonly evaluated for main brain activities. In DMN, the patients with PD in our study had significantly decreased connectivity at the anterior cingulate cortex, superior and medial frontal gyrus, temporal lobe, and precuneus. A previous study found decreased DMN connectivity at similar areas in patients with ESRD (12), without specifying the type of dialysis. DMN is believed to be involved in self-projection and self-awareness (41). The medial prefrontal part is related to episodic memory (42), and the precuneus may be responsible for supporting autobiographical memory and integrating complex information (43). Our findings, which were in agreement with previous studies, demonstrated reduced DMN connectivity, mainly at the medial frontal and precuneus regions. The data indicate that reduced intrinsic brain connections in patients with ESRD can be observed under the condition without clinical cognitive impairment. Furthermore, our patients also had decreased DMN connectivity at the anterior cingulate gyrus, the middle and inferior temporal lobes, which are partially consistent with two earlier reports in patients with ESRD, without mentioning cognitive decline (14, 44). The anterior cingulate cortex is believed to be involved in affect regulation and the ability to manage unpleasant emotions (45). Whether decreased connectivity at these areas is linked to clinical significance requires further longitudinal follow-up.

Interestingly, our DMN data also revealed increased connectivity at the inferior parietal lobule, middle frontal gyrus, supplemental motor area, and insula in the patients, where the supplemental motor area and insula belong to other functional networks such as SMN and SN, respectively. Such cross-network connectivity changes could indicate implicit neurophysiological alterations for sensorimotor or pain perception. For the pain perception, Coppola et al. demonstrated that greater subjective intensity of pain during a migraine attack was associated with weaker DMN-insula connectivity (46), and Usui et al. also presented that the DMN-insula connectivity changes correlated with the difference in pain scores of patients with fibromyalgia after music intervention (47). Chronic pain is common among the ESRD population but is usually underestimated (48). In our hospital, pain assessment was mandatory during an outpatient clinic visit. Although none of the participants in our study reported pain upon enrollment, chronic sensory discomfort was not further evaluated and pain-related activation could not be completely excluded. On the other hand, while the insula and middle frontal gyrus had increased connectivity in the patients with PD in our study, another study showed decreased connectivity in these brain regions in ESRD HD patients (14). The underlying mechanisms of this discrepancy is not fully understood and different renal replacement therapy could play a role. Furthermore, in contrast to the decreased within-DMN connectivity in the patients with ESRD (49), our results denoted the increased DMN-insula connectivity. Based on our experiences, such specific connectivity changes of DMN or SN along with neuropathology could be associated with the alterations (either deteriorative or compensatory) of cognitive performances (39, 50). However, future studies are warranted to verify such inference due to the lack of cognitive tasks in this study.

The current study also found some alterations of SN connectivity in the patient group. SN involves the responses to behaviorally salient events. The patients in our study displayed lower between-network connectivity at the cingulate and postcentral gyri in SN, while insula-thalamus connectivity was clearly increased in the patients with ESRD receiving PD (Table 3), which has never been reported before. In contrast, a recent study that focused on patients receiving HD found hypo-connectivity in the thalamo-cortical network (16). Based on the observations, the SN could be a potential network susceptible to the changes of between-network connectivity in the patients with ESRD receiving PD; however, further investigation is needed to delineate whether dialysis modalities affect the patterns of connectivity alterations.

The patients in our study showed decreased SMN connectivity in the parahippocampal gyrus and insula. In a previous study, decreased SMN has been reported in the precentral and postcentral gyrus (51). SMN is a network associated with sensory input or motor coordination, which is activated by corresponding movement-demanding tasks (52). It is also possibly involved in movement coordination (53). Interestingly, the hippocampus in the patients with ESRD showed a reduced connection to the bilateral SMN, which was rarely observed in this field. Previous papers demonstrated the altered hippocampus-thalamus connection and sensorimotor connectivity separately among neurological and psychiatric patients (54, 55). The reduced hippocampus-SMN connection in the ESRD might be associated with the memory disturbances and slow motor performances in the patients with ESRD, which requires further studies along with memory and movement performances in the future.

In relation to the demographic profiles of our subjects, patients with ESRD had significantly higher uric acid levels when compared to the controls. There is no published data about altered functional connectivity in hyperuricemia subjects. Only one study mentioned the positive correlation between the uric acid level and substantia nigra connectivity (56). In our cohort, no correlation was found between uric acid level and graph metrics (Supplementary Table 3). Uric acid may not play an important role in connectivity change among the patients in our study.

Beyond the connectivity alterations, we performed extra analysis for intrinsic brain activity, the ALFF, which showed no difference between the groups in the current study. Previously, Luo's study (21) showed that patients with ESRD receiving PD had a reduction in ALFF at the inferior parietal lobe. This discrepancy could be related to the clinical profiles. Although the patients in our study did not receive detailed neuropsychological assessment, they did exhibit potent daily functions and good MMSE performance. Patients in Lou's study had cognitive impairments, and their controls did not have any systemic illness. The controls in our study had corresponding diseases as patients with ESRD since underlying chronic illness may influence intrinsic brain activities (33, 34). ALFF may be sensitive to biological changes in the brain activity of patients with ESRD, but this was not evident in our patient group, perhaps because of fair cognitive function and comparative controls.

## Limitations

The main shortcoming of our study was the relatively small sample size. Second, the neuropsychological assessment was incomprehensive, and there was a marginally statistical significance of education years between the two groups. It was difficult to delineate the affected brain regions on connectivity to a respective neuropsychological function domain in the patients in our study. Recruitment of more subjects with a further comparison of functional connectivity between patients with ESRD with different replacement therapies as well as a detailed cognitive assessment should be conducted. Third, a detailed clinical evaluation of pain or paresthesia was not performed. Sensory discomfort should be carefully reviewed in the following ESRD R-fMRI studies. At last, anemia is very frequently observed in patients with ESRD. Literature has shown the influence of hemoglobin on connectivity (57). However, we did not perform the hemoglobin comparison since hemoglobin was not routinely checked for the controls. The possible effect of hemoglobin on connectivity could not be completely excluded.

## Conclusion

We intensively demonstrated the various changes of the brain connectivity in patients with ESRD who underwent PD and preserved favorable cognitive function. In graph analysis, E_glob_, BC, and L were found to be potentially useful metrics to identify the connectivity changes since patients with ESRD showed significantly lower E_glob_ and higher BC and L. Networks including DMN, SMN, SN, and HN all showed alterations in the patients with ESRD, while elevated connectivity strength was observed only in DMN and SN. In patients with ESRD receiving PD, DMN and SN might show more prominent connectivity alterations. Changes in cerebral connectivity may develop without a clinical decline of cognition. Further longitudinal follow-up is required to clarify whether asymptomatic patients with connectivity disruption are prone to have cognitive impairment or dementia later.

## Data Availability Statement

The original contributions presented in the study are included in the article/Supplementary Material, further inquiries can be directed to the corresponding author/s.

## Ethics Statement

The studies involving human participants were reviewed and approved by Institutional Review Board of Chang Gung Memorial Hospital (IRB Approval Number: 102-1346A3). The patients/participants provided their written informed consent to participate in this study.

## Author Contributions

T-YC, H-HW, and CW: manuscript writing. T-YC, C-HY, and H-SJ: data acquisition. H-LL, H-SJ, and CW: data analysis. T-YC, H-HW, Y-JL, K-LH, T-HL, and Y-CT: data interpretation and literature review. H-LL, T-HL, Y-CT, and CW: research mentorship and supervision. All authors contributed important intellectual content during manuscript drafting or revision and accepts accountability for the overall work by ensuring that questions pertaining to the accuracy or integrity of any portion of the work are appropriately investigated and resolved.

## Funding

This work was supported by two grants (CMRPG3G0271 and CMRPG3A1041) from the Chang Gung Medical Research Council. The funders had no role in the study design or interpretation of the finding.

## Conflict of Interest

The authors declare that the research was conducted in the absence of any commercial or financial relationships that could be construed as a potential conflict of interest.

## Publisher's Note

All claims expressed in this article are solely those of the authors and do not necessarily represent those of their affiliated organizations, or those of the publisher, the editors and the reviewers. Any product that may be evaluated in this article, or claim that may be made by its manufacturer, is not guaranteed or endorsed by the publisher.
