# Supplementary material for: Changes of Brain Functional Connectivity in End-Stage Renal Disease Patients Receiving Peritoneal Dialysis Without Cognitive Decline
Source: Front Med (Lausanne). 2021 Nov 24;8:734410. doi: 10.3389/fmed.2021.734410 (PMC8652044; doi:10.3389/fmed.2021.734410)
Supplement: Supplementary file 1 [file Data_Sheet_1.docx]

***Supplementary Material***

**Table S1.** **Anatomical ROIs generated based on the AAL parcellation. Each of the 45 labels contains ROIs in both left and right hemispheres, which yield a total of 90 ROIs.**

| Precentral gyrus | Lingual gyrus |
| --- | --- |
| Superior frontal gyrus (dorsal) | Superior occipital gyrus |
| Orbitofrontal cortex (superior) | Middle occipital gyrus |
| Superior frontal gyrus (medial) | Inferior occipital gyrus |
| Orbitofrontal cortex (medial) | Fusiform gyrus |
| Middle frontal gyrus | Postcentral gyrus |
| Orbitofrontal cortex (middle) | Superior parietal gyrus |
| Inferior frontal gyrus (opercula) | Inferior parietal lobule |
| Inferior frontal gyrus (triangular) | Supramarginal gyrus |
| Orbitofrontal cortex (inferior) | Angular gyrus |
| Rolandic operculum | Precuneus |
| Supplementary motor area | Paracentral lobule |
| Olfactory | Caudate |
| Rectus gyrus | Putamen |
| Insula | Pallidum |
| Anterior cingulate gyrus | Thalamus |
| Middle cingulate gyrus | Heschl gyrus |
| Posterior cingulate gyrus | Superior temporal gyrus |
| Hippocampus | Temporal pole (superior) |
| Parahippocampal gyrus | Middle temporal gyrus |
| Amygdala | Temporal pole (middle) |
| Calcarine cortex | Inferior temporal gyrus |
| Cuneus |  |

**
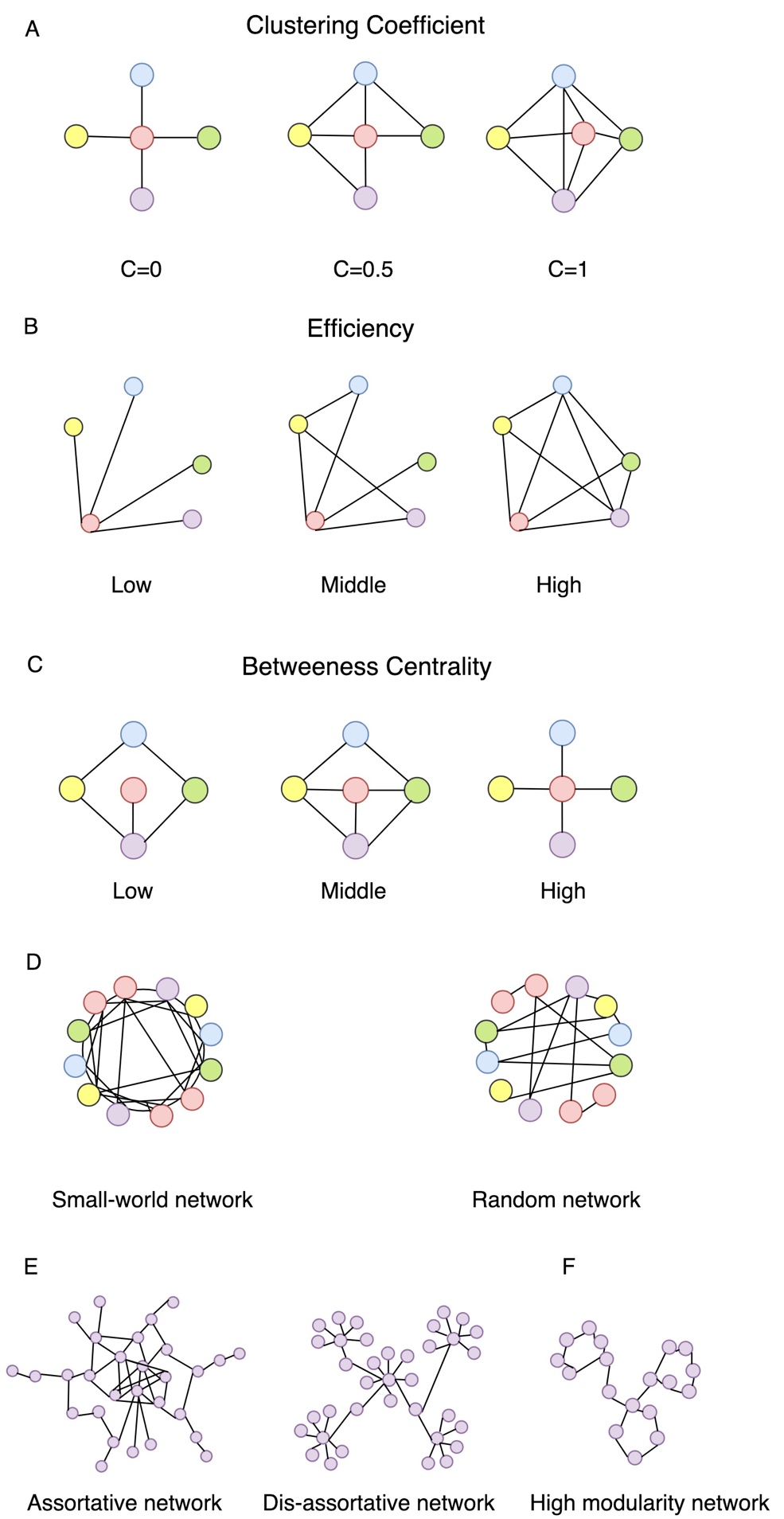
**

**Figure S1.** Topological parameters of graph theory: (A) Clustering Coefficient: a measure of degree to which nodes in a graph tend to cluster together; (B) Efficiency: the average inverse shortest path length in the network; (C) Betweeness Centrality: the fraction of all shortest paths in the network that contain a given node; (D) Small-world network: a class of network that is highly clustered, yet has small characteristic path lengths, like random networks; (E) Assortativity: how often nodes with a certain degree are linked to nodes with a similar degree; (F) Modularity: a subset of vertices in a graph that have higher connections with each other than with the rest of the network.

**The mathematical definitions of graph network parameters**:

1. For a given graph N composed of n nodes, the **Degree** of a node “i” is defined as:

$$k_{i}=\sum_{j\in N} a_{\mathrm{ij}}$$

where a_ij_ is the connection status between node i and j: a_ij_ = 1 if link exist, otherwise a_ij_ = 0.

1. The **Clustering coefficient** represents how many nearest neighbors of node i are connected to each other as well, and is defined as [^1^](#_ENREF_1):

$$C_{i}=\sum_{i\in N} \frac{2t_{i}}{k_{i}(k_{i}-1)means}$$

where t_i_ is the number of triangles around a node i.

1. The **Local efficiency** represents the efficiency of the local sub-graph of a node i that contains only the direct neighbors of node i, and is defined as [^2^](#_ENREF_2):

$$E_{loc,i}=\frac{\sum_{j,h\in N, j\neq i} a_{\mathrm{ij}}a_{\mathrm{ih}}\left[ d_{\mathrm{jh}}(N_{i}) \right]^{-1}}{k_{i}(k_{i}-1)}$$

where d_jh_(N_i_) is the length of the shortest path between j and h which contains only neighbors of i.

1. The **Betweenness centrality** is the fraction of all shortest paths in the network that pass through a given node i and is defined as [^3^](#_ENREF_3):

$$b_{i}=\sum_{\begin{aligned} h,j\in N \\ h\neq j, h\neq i, j\neq i \end{aligned}} \frac{\rho_{\mathrm{hj}}(i)}{\rho_{\mathrm{hj}}}$$

where ρ_hj_ is the number of shortest paths between h and j, and ρ_hj_(i) is the number of shortest paths between h and j that pass through i.

1. The **Characteristic path length** is the average of all shortest paths between each possible pair in the network and defined as [^1^](#_ENREF_1):

$$L=\frac{1}{n}\sum_{i\in N} L_{i}=\frac{1}{n}\sum_{i\in N} \frac{\sum_{j\in N, j\neq i} d_{\mathrm{ij}}}{n-1}$$

where d_ij_ is the shortest path length (distance) between nodes i and j, and L_i_ is the average distance between node i and all other nodes.

1. The **Global efficiency** is the average inverse shortest path length and defined as [^2^](#_ENREF_2):

$$E=\frac{1}{n}\sum_{i\in N} E_{i}=\frac{1}{n}\sum_{i\in N} \frac{\sum_{j\in N, j\neq i} d_{\mathrm{ij}}^{-1}}{n-1}$$

where E_i_ is the efficiency of node i.

1. To estimate the property of network Small-worldness, a matched random network with same number of nodes and edges as the real network was generated.

The **Small-worldness** represents the network with a higher local clustering but nearly the same characteristic path length as random networks, and is defined as the ratio of normalized clustering coefficient (γ=C / C_rand_) and normalized characteristic path length (λ=L / L_rand_) [^1^](#_ENREF_1):

$$\sigma=\frac{C/{C_{\mathrm{rand}}}}{L/{L_{\mathrm{rand}}}}$$

where C and L are the average Clustering coefficient and the Characteristic path length of real networks, and C_rand_ and L_rand_ indicate the means of random networks. In general, the Small-world topological organization should meet the criteria of γ > 1 and λ ≈ 1, or σ = γ / λ > 1 [^4^](#_ENREF_4).

1. The **Modularity** is a subset of vertices in a graph that have higher connections with each other than with the rest of the network and is defined as [^5^](#_ENREF_5):

$$Q=\sum_{u\in M} \left[ e_{\mathrm{uu}}-\left( \sum_{v\in M} e_{\mathrm{uv}} \right)^{2} \right]$$

where the network is fully segmented to a set of non-overlapping modules “M”. The modules u and v are the subsets in modules M, and e_uv_ is the number of all links that connect nodes in module u with nodes in module v.

1. The **Assortativity coefficient** means how often nodes with a certain degree are linked to nodes with a similar degree and is defined as [^6^](#_ENREF_6):

$$r=\frac{l^{-1}\sum_{(i,j)\in L} k_{i}k_{j}-\left[ l^{-1}\sum_{(i,j\in L)} \frac{1}{2}\left( k_{i}+k_{j} \right) \right]^{2}}{l^{-1}\sum_{(i,j)\in L} \frac{1}{2}\left( k_{i}^{2}+k_{j}^{2} \right)-\left[ l^{-1}\sum_{(i,j)\in L} \frac{1}{2}\left( k_{i}+k_{j} \right) \right]^{2}}$$

where l is the number of links between i and j.

**Amplitude of Low-Frequency Fluctuation (ALFF)**

After the preprocessing steps, we also calculated the square root of power spectrum of the R-fMRI temporal fluctuations, which is a marker for the intrinsic brain activity intensity in a voxel-wise manner. ALFF calculation was carried out using the REST toolkit, and the frequency range was set within 0.01-0.08 Hz (removing the very-low-frequency drift and high-frequency noise). However, the ALFF showed no significant difference of signal activation between the ESRD patients and the controls (Figure.S2).

**
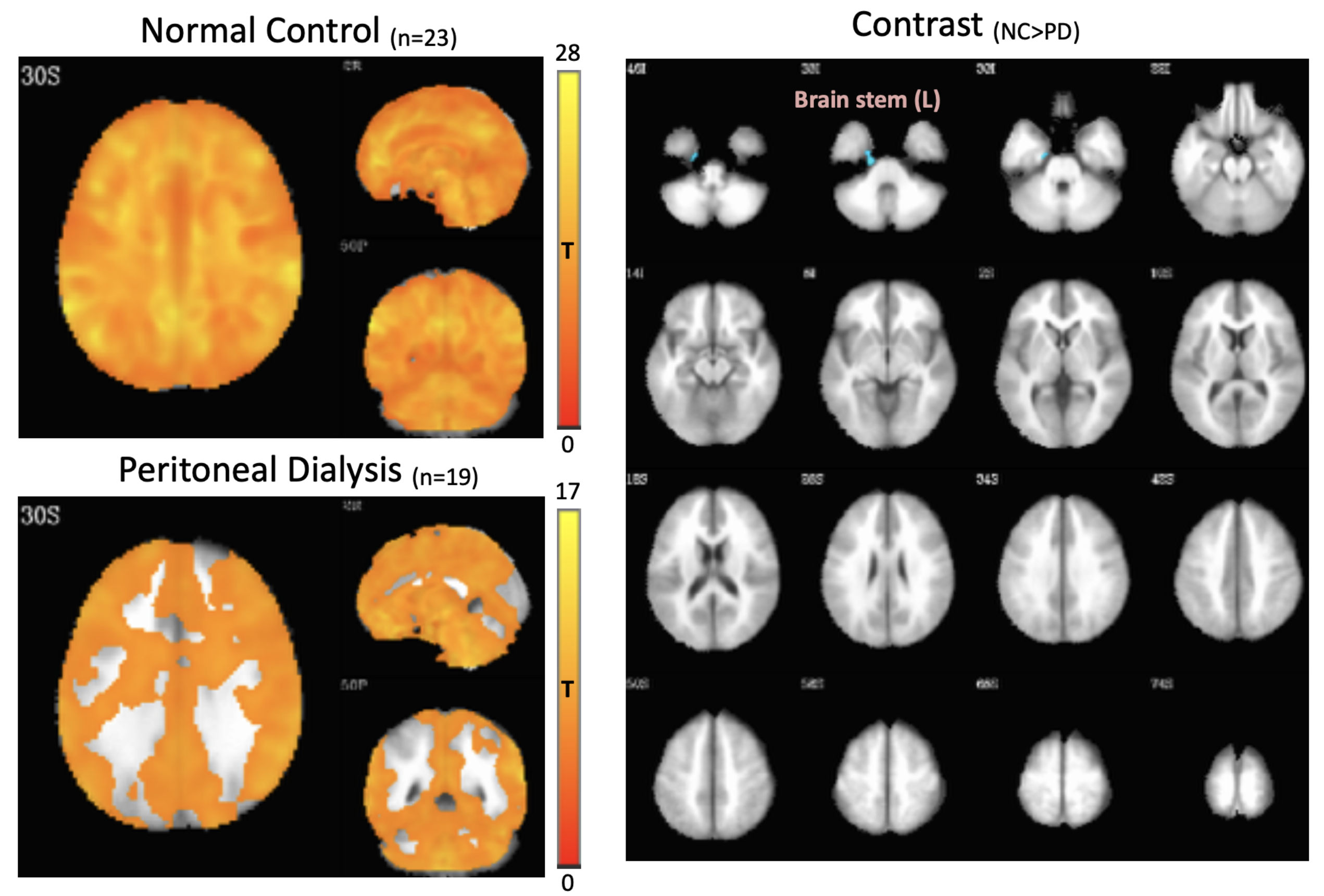
**

**Figure S2.** Amplitude of low-frequency fluctuations (ALFF) map of patients and controls. The connectivity map (FWE *p* < 0.05, cluster = 20 voxels) of either group is shown at the left side, and the contrast map (3dClustSim-corrected *p* < 0.05) at the right side. A trend of decreasing connectivity in PD patients is observed in the map (the left row), but no obvious significance is seen compared with controls (the right row, contrast map).

**Table S2. Duration of receiving peritoneal dialysis (PD) therapy for each end stage renal disease (ESRD) patient**

| Patient | Gender | Age | Duration  (years) |
| --- | --- | --- | --- |
| 1 | M | 41 | 1 |
| 2 | M | 47 | 6 |
| 3 | F | 70 | 1 |
| 4 | M | 30 | 1 |
| 5 | M | 75 | 9 |
| 6 | M | 34 | 2 |
| 7 | F | 48 | 7 |
| 8 | F | 65 | 2 |
| 9 | F | 26 | 2 |
| 10 | M | 49 | 6 |
| 11 | M | 58 | 2 |
| 12 | M | 41 | 16 |
| 13 | F | 37 | 3 |
| 14 | F | 59 | 2 |
| 15 | M | 51 | 3 |
| 16 | F | 56 | 7 |
| 17 | F | 55 | 4 |
| 18 | F | 51 | 5 |
| 19 | F | 53 | 1 |

**M, male; F, female.**

**Table S3. Correlation Between Uric Acid and Graph Analysis Metrics**

| **Group** | **Lab data vs. Graph Metrics** | ***r* (95% CI)** | **R^2^** | ***p*** |
| --- | --- | --- | --- | --- |
| PD | Uric Acid vs. Betweenness Centrality | <0.01 (-0.45 to 0.45) | <0.01 | 0.98 |
|  | Uric Acid vs. Characteristic Path Length | -0.14 (-0.56 to 0.34) | 0.02 | 0.58 |
|  | Uric Acid vs. Global Efficiency | 0.29 (-0.19 to 0.66) | 0.08 | 0.23 |
| All | Uric Acid vs. Betweenness Centrality | -0.11 (-0.40 to 0.19) | 0.01 | 0.47 |
|  | Uric Acid vs. Characteristic Path Length | -0.02 (-0.31 to 0.29) | <0.01 | 0.92 |
|  | Uric Acid vs. Global Efficiency | -0.20 (-0.47 to 0.10) | 0.04 | 0.19 |

PD, peritoneal dialysis; *r*, Pearson correlation coefficient; CI, confident interval;

All, peritoneal dialysis patients plus controls
